# Healthcare interpreter utilisation: analysis of health administrative data

**DOI:** 10.1186/s12913-018-3135-5

**Published:** 2018-05-10

**Authors:** Nicole Blay, Sharelle Ioannou, Marika Seremetkoska, Jenny Morris, Gael Holters, Verily Thomas, Everett Bronwyn

**Affiliations:** 1 Centre for Applied Nursing Research (CANR), Ingham Institute, SWSLHD, Locked Bag 7103, Liverpool BC, NSW 1871 Australia; 2 School of Nursing and Midwifery, Western Sydney University, Locked Bag 1797, Penrith, NSW 2751 Australia; 3 Bankstown-Lidcombe Hospital, Eldridge Road, Bankstown, NSW 2200 Australia

**Keywords:** CALD, Administrative data, Healthcare, Interpreter, limited English proficiency, Language, Obstetrics

## Abstract

**Background:**

Few people with limited English proficiency are provided with the services of a healthcare interpreter when admitted to hospital. This retrospective study utilised health administrative data to explore which patients with limited English proficiency were provided with a healthcare interpreter during their hospital admission.

**Method:**

A retrospective analysis of health administrative data for adult overnight-stay patients admitted to a public hospital in a region of significant cultural and linguistic diversity in Sydney, Australia in 2014–2015. Descriptive analyses were used to explore demographic and diagnostic data. Chi-square and analysis of variance were used to test for association between variables.

**Results:**

The site hospital provided for 19,627 overnight-stay episodes of care over the one year period. Emergency admissions made up 70.5% (*n* = 13,845) of all hospital admissions and obstetric patients 11.7% (*n* = 2291). For 15.7% (*n* = 3074) of episodes of care a healthcare interpreter was identified at hospital admission as being required. In 3.7% (*n* = 727) of episodes of care a healthcare interpreter was provided. Patients who received an interpreter were more likely to be female, of a younger age and admitted to hospital for childbirth.

**Conclusions:**

A minority of patients with limited English proficiency received a healthcare interpreter during their episode of care. The majority of interpreter services were provided to obstetric patients.

## Background

Healthcare interpreters have been found to enhance communication between healthcare providers and patients with limited command of the English language. The provision of an interpreter for individuals with limited English proficiency (LEP) defined as speaking English less than ‘very well’ [1], is important in terms of patient safety [2] by increasing communication, health literacy and comprehension [3, 4]; ongoing nursing and medical management; self-care efficacy and access to services [4–7].

In Australia and elsewhere, the utilisation of healthcare interpreters for LEP patients varies with reported rates ranging from less than 1% to 97% of LEP patient consultations [8–13]. The situation is no different within New South Wales (NSW) including the Local Health District (LHD) where this study was conducted. In 2013–2014, 16% of hospital inpatients received the interpreter service compared to 71% of hospital outpatients [14]. This is concerning when it is considered that 48% of the local population speak a language other than English at home and have poorer health outcomes compared to the NSW average [14–16].

To aid communication, healthcare practitioners most often rely on informal interpreters including other healthcare providers fluent in the required language, and/or family members [8]. Reasons for selecting informal interpreters over healthcare interpreters include the need to book a healthcare interpreter as opposed to immediate access to family members; availability of healthcare interpreters; and health practitioners’ time constraints [2, 8, 11, 17–20]. Additional reasons included confidentiality; familiarity with the patient and family members; confidence in one’s own language skills; difficulty in assessing the need for an interpreter and a lack of knowledge of the interpreter service or booking system [8, 11, 17–19].

Patient preference for and satisfaction with a healthcare interpreter compared to informal interpreter(s) is mixed. Some patients with cancer had a preference for interpretation by family members [21] whereas the healthcare interpreter was preferred by those whose condition was of a ‘personal nature’ [22]. Results from a systematic review found little difference in patient satisfaction between the use of bilingual clinicians compared to healthcare interpreters [23] while Emergency Department patients reported higher levels of satisfaction when interpretation was provided by healthcare interpreters compared to informal interpreters [24]. It is therefore possible that the booking and provision of a healthcare interpreter is to some extent, dependent on patients’ clinical conditions and personal preferences.

As research on healthcare interpreter utilisation has primarily focussed on specific patient populations [6–9, 12] and/or language groups [5, 6, 9, 25], the characteristics of inpatients for whom the healthcare interpreter service is provided, remains largely unknown. Based on diagnostic data, this study sought to explore which patients were provided with a healthcare interpreter in the hospital setting.

## Method

### Aim

To retrospectively examine healthcare databases to determine which type of patient with limited English proficiency admitted to a metropolitan hospital received the services of a healthcare interpreter.

### Design

This retrospective, descriptive study formed a component of a larger research project exploring the impact of healthcare interpreter provision on hospital-related outcomes. Merged health administrative and interpreter datasets were retrospectively analysed for all overnight-stay patients admitted to the site hospital over a 12 month period in 2014–2015.

### Setting

Adult patients admitted for an overnight stay to a 434 bed public principal referral hospital in Sydney, NSW in one of the most culturally and linguistically diverse regions in Australia [26]. During the period of study, a total of 47,500 day-only (not specified) and overnight-stay patients were admitted to the site hospital [27].

### Procedure

Health administrative and inpatient data were used for this study. De-identified inpatient data for the 2014–2015 Financial Year were extracted and downloaded in Microsoft Excel format from the NSW Health repository, Health Information Exchange (HIE). Interpreter service provisions were obtained from the independent Interpreter and Translating Service database for the same period, for the site hospital. The two datasets were merged based on the Medical Record Number and logical formulae to ensure that only healthcare interpreter services provided during an inpatient episode at the site hospital were included. Variables in the merged dataset included patient demographics, patient admission variables, diagnosis related groups (DRGs), interpreter required, interpreter booked and service provisions. The ‘Interpreter Required’ box completed on admission to hospital was used as a proxy indicator for limited English proficiency. Data were not available for informal interpretations as provided by bilingual healthcare practitioners and/or other carers.

#### Analyses

SPSS Version 23.0 [28] was used for data analysis. Descriptive statistics were used to portray categorical data including type of admission and patient demographic data. Chi-square was used to test for association between categorical values. For analysis purposes data were dichotomised for admission type (emergency and all other admissions). Independent t-test and analysis of variance were used to compare distributions across groups. All statistical analyses were performed at the 0.05 significance level.

## Results

The merged databases represented 19,627 inpatient episodes of care^1^ and 14,977 individual patients, indicating that almost 20% of patients were admitted to hospital more than once during the study period. Emergency admissions represented the vast majority of episodes of care (70.5%, *n* = 13,845), planned (elective) admissions made up 16.7% (*n* = 3287) and obstetric patients represented 11.7% (*n* = 2291) episodes of care. A minority of episodes of care were unassigned or coded as day-only admissions.

### Healthcare interpreter provision

**Fig. 1:**
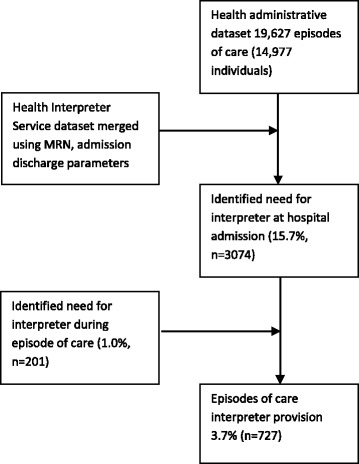
Flow diagram demonstrating method

A need for an interpreter was identified at hospital admission in 15.7% (*n* = 3074) episodes of care with 526 (17.1%) of these patients going on to receive the healthcare interpreter service. Overall, the healthcare interpreter was provided in 3.7% (*n* = 727) of episodes of care, including 201 (1%) patients, whose need for an interpreter was not identified at hospital admission but at a later point during their episode of care (refer to Fig. 1 Flow chart showing the research method and results). It is not known if the healthcare practitioner identified the need for an interpreter or if the service was requested by the patient or family member.

The majority of interpreter provisions were provided to emergency admissions (68.2%, *n* = 496), 23.4% (*n* = 170) to obstetric patients and 7.0% (*n* = 51) to planned admissions. Analysis of variance demonstrated a significant difference between interpreter provision and admission type ANOVA F(13,713) =2.512, *p* = .002).

Healthcare interpreters provided on average 2.3 (SD 2.55, range 1–35) sessions per episode of care. However, for 52.4% (*n* = 381) of episodes of care, only one session was provided. Healthcare interpreter services provided for emergency (*n* = 496) and all other admission types (*n* = 231) were examined. Emergency patients received on average 1.96 (SD 2.063) healthcare interpreter services compared to 1.42 (SD 1.439) interpreter services for all other patient admission types. This was significant at the .05 level, ANOVA F(13,713) = 3.035, *p* < .001).

### Patient demographics

Patients who received the interpreter service were predominantly female (64.6%, *n* = 470) with an average age of 56.7 years, which was significantly younger than males (72.5 years) (*p* = .000). They were born in 51 different countries and spoke 41 different languages with Arabic, Vietnamese and English being the most frequently recorded languages (refer to Table 1). Patients recorded as English speaking (*n* = 79) were born in 29 different countries: principally Lebanon (17.7%, *n* = 14) followed by Vietnam, China and Australia at 12.7% (*n* = 10) respectively.

**Table 1:** Top 10 languages and interpreter provision

Recorded language and interpreter provision		
	n	%
Arabic (including Lebanese)	225	30.9
Vietnamese	156	21.5
English	79	10.9
Greek	49	6.7
Mandarin	35	4.8
Cantonese	33	4.5
Macedonian	28	3.9
Italian	18	2.5
Chinese (no further details)	17	2.3
Croatian	9	1.2

### Principal diagnoses

The principal diagnosis for each episode of care was examined for patients who received a healthcare interpreter. Demonstrating the diversity in the patient base, 308 principal diagnoses were identified across 695 episodes of care.

As shown in Table 2, the majority (21%, *n* = 150) of healthcare interpreter services were provided to obstetric patients. Childbirth related diagnoses were responsible for the top two principal diagnoses and also equal third with cerebral infarction for interpreter provision. Overall, healthcare interpreter services were provided to 170 episodes of care with an obstetric-related principal diagnosis. Fewer patients with congestive cardiac failure, urinary tract infection and chronic obstructive pulmonary disease received an interpreter. In light of females and obstetric patients being the major recipients of the interpreter service; obstetric patients were removed from the database and further analyses conducted. Females represented 56% (*n* = 1512) of this sample (*χ*^2^ p = <.001) however, a non-significant result was returned for gender and interpreter provision (*t* (2667) = .211, *p* = .833).

**Table 2:** Top 10 principal diagnoses and healthcare interpreter provision

Principal Diagnosis			
Code	Description	n	%
O80	Single spontaneous delivery	101	14.5
O82	Single delivery by caesarean section	33	4.7
O81	Single delivery by forceps and vacuum extractor	16	2.3
I63.9	Cerebral infarction unspecified	16	2.3
I50.0	Congestive heart failure	14	2.0
N39.0	Urinary tract infection, site not specified	13	1.9
J44.0	Chronic obstructive pulmonary disease with acute lower respiratory infection	10	1.4
A41.9	Fracture of intertrochanteric section of femur	8	1.2
S72.11	Sepsis, unspecified	8	1.2
J69.0	Pneumonitis due to food and vomit	7	1.0
	All other diagnoses	535	73.6

## Discussion

This study has utilised routinely collected healthcare data to explore which overnight-stay patients were provided with a healthcare interpreter during their hospital episode. Consistent with published global literature few LEP patients received a healthcare interpreter during their episode of care. However, it is not known whether the number of patients identified as needing the healthcare interpreter service is representative of need. Less than 16% of patients who had been identified as needing a healthcare interpreter at hospital admission were provided with the service while a minority of patients considered to be proficient in the English language at hospital admission, went on to require the service. It is possible that informal interpreters such as bilingual clinicians and/or family members were utilised but data on the incidence of informal interpretations is not collected in NSW. It is likely that many informal interpretations are spontaneous responses to patients and/or family members’ queries and from a need to keep patients informed of their management and care.

Of interest is the 11% of patients who received a healthcare interpreter who were recorded in the medical record as English speaking. Such data were collected from the patient and/or family members at hospital admission; it is therefore possible that some individuals considered themselves to be proficient in the English language but that their language skills were not at a level necessary for the communication of clinical outcomes. For some LEP individuals, being identified as English speaking is perceived as a sign of social standing and cultural assimilation [29–31] while others feel obliged to use a healthcare interpreter despite confidence in their own English language skills [31]. Indeed, several studies have indicated that self-reported and practitioner assessment of language proficiency, as occurs in healthcare, is not always reliable [18, 29, 30]. Finally, the possibility of data error cannot be excluded considering that a minority of English-speaking patients who received the healthcare interpreter were reportedly born in Australia.

The study found that women admitted to hospital for obstetric services made up the largest percentage of those who received a healthcare interpreter during their episode of care. The high rate of healthcare interpreter provision for the obstetric population was unexpected. Based on 70% of episodes of care being categorised as an emergency admission, it would not be unreasonable to anticipate that the majority of healthcare interpreter services would be provided to patients in the acute sector; however it is also acknowledged that the diversity of diagnoses may have skewed results. A concerning finding was that few elective patients whose admission to hospital was a planned event, received the interpreter. Having a planned admission to hospital allows for the interpreter to be booked in advance. However, it is recognised that some such patients may have been provided with an interpreter at pre-admission; a time period beyond the scope of this research project.

Definitive data are not available, but it is possible that a proportion of the women receiving interpreters were refugees. In the geographical region serviced by the study hospital, 41% of individuals have settled under the humanitarian (refugee) program [14]. This is important as the majority of female refugees are under the age of 26 years [32] and 44% have no understanding of English prior to arriving in Australia [33]. At a clinical level, it is essential that midwives and other healthcare providers are able to communicate with labouring women and their partners to ensure safety during childbirth, and competency and confidence with newborn feeding and care [34–36]. Obstetric patients with LEP received at least one healthcare interpreter session, although it is not known at what stage of the episode of care this was provided. Considering that only 50% of women from culturally and linguistically diverse backgrounds are offered an interpreter during labour [37] it is likely that the healthcare interpreter was provided for postnatal education and care. Anecdotal evidence supports this theory with reports that interpreter bookings are made for small groups of patients speaking the same language. Further research is necessary to determine if this is the case.

### Limitations

As a retrospective study utilising health administrative and interpreter datasets, the study has some limitations. The accuracy of data is unable to be verified and reasons behind the low level of interpreter service provision are unable to be determined. However, the study has provided insight into which patients are more likely to receive the healthcare interpreter service. A prospective study is recommended to explore reasons for the low level of interpreter provision, and any demographic differences between patients.

## Conclusion

This study has determined that a minority of LEP patients admitted to hospital in a region of significant cultural and linguistic diversity receive a healthcare interpreter. Females were more likely than males to receive an interpreter, reflecting the high proportion of obstetric patients. Further research is required to explore the characteristics of obstetric patients receiving interpreter assistance and the incidence of small group interpreter services in the obstetric population.

## Abbreviations

DRGs: Diagnosis related groups
HIE: Health Information Exchange
LEP: Limited English proficiency
LHD: Local Health District
NSW: New South Wales

## Footnotes

1 Episode of care in this study is equivalent to a hospital admission.
